# Degradation of Tyrosine Hydroxylase by the Ubiquitin-Proteasome System in the Pathogenesis of Parkinson’s Disease and Dopa-Responsive Dystonia

**DOI:** 10.3390/ijms21113779

**Published:** 2020-05-27

**Authors:** Ichiro Kawahata, Kohji Fukunaga

**Affiliations:** Department of Pharmacology, Graduate School of Pharmaceutical Sciences, Tohoku University, Sendai 980-8578, Japan

**Keywords:** Parkinson’s disease, dopa-responsive dystonia, tyrosine hydroxylase, α-synuclein, fatty acid-binding protein 3, ubiquitination, proteasomal degradation, ubiquitin-proteasome system

## Abstract

Nigrostriatal dopaminergic systems govern physiological functions related to locomotion, and their dysfunction leads to movement disorders, such as Parkinson’s disease and dopa-responsive dystonia (Segawa disease). Previous studies revealed that expression of the gene encoding nigrostriatal tyrosine hydroxylase (TH), a rate-limiting enzyme of dopamine biosynthesis, is reduced in Parkinson’s disease and dopa-responsive dystonia; however, the mechanism of TH depletion in these disorders remains unclear. In this article, we review the molecular mechanism underlying the neurodegeneration process in dopamine-containing neurons and focus on the novel degradation pathway of TH through the ubiquitin-proteasome system to advance our understanding of the etiology of Parkinson’s disease and dopa-responsive dystonia. We also introduce the relation of α-synuclein propagation with the loss of TH protein in Parkinson’s disease as well as anticipate therapeutic targets and early diagnosis of these diseases.

## 1. Introduction

Parkinson’s disease (PD) is a common disease whose prevalence is increasing owing to the aging society. PD is clinically characterized by movement disabilities, such as resting tremor, rigidity, and bradykinesia [1]. PD is also defined pathologically by the selective degeneration of dopaminergic neurons in the substantia nigra pars compacta (SNpc) and by the cytoplasmic accumulation of proteinaceous inclusions, termed Lewy bodies [2,3]. Dopa-responsive dystonia (DRD), also termed as Segawa disease, is a disorder that involves involuntary muscle contractions, tremors, and other uncontrolled movements, which usually appear during childhood [4]. DRD patients present with reduced nigrostriatal dopaminergic function [5,6]. As widely known, PD and DRD are neurodegenerative disorders that predominately affect midbrain dopamine-producing neurons. Though dysfunctions of the dopaminergic system are involved in neurological disorders, such as Tourette’s syndrome [7], schizophrenia [8,9], pituitary tumors [10], PD [11,12,13,14,15], and DRD [4,5,16], the loss of nigrostriatal tyrosine hydroxylase (TH) protein is distinctive in PD and DRD. The etiology of PD and DRD has been studied in the past quarter-century; however, the molecular mechanism of the onset of the disorders has not been completely elucidated. In particular, the reason why the TH protein, which is a rate-limiting enzyme of dopamine biosynthesis, is lost in mesencephalic dopaminergic neurons in PD and DRD, and is not entirely understood. In this review, we focus on the molecular mechanism of the loss of TH protein in the neurodegeneration process in PD and DRD by introducing the degradation of phosphorylated TH protein through the ubiquitin-proteasome system. We also introduce the relation between the loss of TH protein and the propagation of α-synuclein, which is a well-known protein in PD pathology, to clarify the mechanism underlying the reduction of nigrostriatal dopamine function and the loss of TH protein in these movement disorders.

## 2. Pathology of Parkinson’s Disease and Dopa-Responsive Dystonia

PD was first diagnosed and described in detail by James Parkinson in 1817 [1]. PD affects over 10 million worldwide, particularly 1%–3% of the global population aged over 60 years and up to 50% of individuals aged over 85 [17]. The clinical features of PD are resting tremor, rigidity, bradykinesia, gait disturbances, postural instability [1], and dementia, which becomes common in the advanced stage of the disease [18]. Pathologically, PD is characterized by the loss of dopamine-biosynthesizing neurons in the substantia nigra pars compacta (SNpc), and by the abnormal deposition of α-synuclein in the cell body (called Lewy body) and in neuronal processes (called Lewy neurites). The risk of developing PD is twice as high in men than in women; particularly, women have a higher mortality rate and faster progression of the disease [19]. Moreover, 90% of PD are sporadic, and hereditary and environmental factors are thought to be involved in the etiology of PD. Currently, over 20 causative or putative genes of hereditary PD have been identified by genetic linkage analysis [20]; for example, *SNCA* (*PARK1*, *PARK4*), *Parkin* (*PARK2*), *DJ-1* (*PARK7*), and *LRRK2* (*PARK8*) [21,22,23,24,25,26,27], which encode α-synuclein, Parkin, protein/nucleic acid deglycase DJ-1, and leucine-rich repeat kinase 2 (LRRK2) protein, respectively. These different gene mutations in familial PD point to the possibility that an alteration in protein conformation and/or degradation could be a key to the degenerative process.

Another dopaminergic disorder, dystonia, is a heterogenous, neurological disorder characterized by abnormal involuntary sustained muscle contractions, frequently causing twisting and repetitive movements or abnormal postures [28]. It is believed that approximately 70% of all patients with dystonia have idiopathic rather than symptomatic dystonia. The mechanisms of dystonia pathogenesis include abnormalities in the regulation of dopaminergic transcription, nigrostriatal dopamine signaling, and loss of inhibition at neuronal circuits. There are at least 11 different genes involved in autosomal dominant inherited dystonia, one in autosomal recessive inherited dystonia, and another in X-linked recessive inherited dystonia [29]. One of the most common genetic dystonia, dopa-responsive dystonia (DRD, *DYT5*), is mainly caused by the mutation of *GCH1* [4,30], which encodes GTP cyclohydrolase 1 (GCH1). Women are more commonly affected, with men showing a lower penetrance of mutations [31,32]; this disease develops in early childhood at approximately age 5–8 [4].

In common, PD and DRD are associated with impaired nigrostriatal dopaminergic function [33]. Nigrostriatal dopaminergic projections play a central role in the control of voluntary movements, and their degeneration has been implicated in Parkinsonian clinical symptoms. In addition, the dopaminergic system, originating in the SNpc and the ventral tegmental area (VTA), which mainly projects to the striatum (mesostriatal pathway) and the prefrontal context (mesocortical pathway), plays a major motivational role in behavioral actions [34,35,36]. Consistently, lesions in nigral neurons lead to simultaneous dysfunction of agonist and antagonist muscle pairs in animal models of parkinsonism [37] and idiopathic PD [15]. The dopaminergic function is regulated by dopamine, which is biosynthesized from L-tyrosine by TH and aromatic L-amino acid decarboxylase (AADC). TH requires tetrahydrobiopterin, which is biosynthesized by GCH1, to perform its enzymatic activity. Because the enzymatic activity of TH protein strictly controls the rate-limiting step of dopamine biosynthesis, unlike those of other dopamine biosynthesizing enzymes, the expression level and activity of TH directly affect intracellular dopamine amount. Thus, we next focus on the physiological features of TH protein and its implications in PD and DRD pathogenesis.

## 3. Physiology of Tyrosine Hydroxylase Phosphorylation

TH is a rate-limiting enzyme for dopamine biosynthesis [38] and is selectively expressed in monoaminergic neurons in the central nervous system. In humans, TH protein has four isoforms with different molecular weight, which are derived from the same gene through alternative splicing of mRNA [39,40]. This protein also has two isoforms in monkeys and only a single isoform in all nonprimate mammals [41,42]. The catalytic domain of TH is located within the C-terminal area, whereas the region that controls enzyme activity (the regulatory domain) is located at the N-terminal end [43]. Four phosphorylation sites, namely Ser8, Ser19, Ser31, and Ser40, have been identified in the N-terminal region of TH [44], whereas the catalytic domain is in 188–456 amino acid residue [45]. TH is a homotetramer consisting of four subunits, and the C-terminal domain forms this homotetramer structure [46].

Two mechanisms can modulate the activity of TH: one is a medium- to long-term regulation of gene expression, such as enzyme stability, transcriptional regulation, RNA stability, alternative RNA splicing, and translational regulation. The regulation of TH is well known; its expression level depends on transcription driven by cyclic adenosine monophosphate (cAMP)-dependent responsive element (in promoter) [47] in a manner dependent on activator protein 1 (AP-1) [48,49], serum-responsive factor (SRF) [50], and nuclear receptor related-1 (Nurr1) [51]. The other is a short-term regulation of enzyme activity, such as feedback inhibition, allosteric regulation, and phosphorylation [47,52,53]. Many factors strictly regulate the activity of TH to control dopamine biosynthesis. Upon depolarization, cyclic AMP-dependent protein kinase (PKA) and calcium-calmodulin-dependent protein kinase II (CaMKII) are activated [54,55,56]. PKA phosphorylates TH at Ser40 and CaMKII phosphorylates TH at Ser19 [57,58]. Phosphorylation of Ser19 increases Ser40 phosphorylation, indicating that the phosphorylation of Ser19 can potentiate the phosphorylation of Ser40 and subsequent activation of TH [59]. Other stress-related protein kinases can also phosphorylate TH at Ser40 [52,53]. Phosphorylation at Ser40 leads to the liberation of dopamine from the active site of TH and changes the conformation to the high specific activity form [60]. Cytosolic free dopamine can bind to the active site of TH and deactivate the enzyme to suppress dopamine overproduction [61,62]. It has been reported that the phosphorylated form of TH is highly labile, whereas the dopamine-bound form is stable [63]. TH phosphorylated at Ser40 (pSer40-TH) is dephosphorylated by a protein phosphatase, such as protein phosphatase 2A (PP2A), because inhibition of PP2A with okadaic acid or microcystin induces an increase in pSer40-TH level [64,65,66]. Ser31 phosphorylation is mediated by extracellular signal-regulated kinase 1 (ERK1) and ERK2 [42,67], and its dephosphorylation is mediated by PP2A [66]. Because ERK signals are usually activated as part of the mitogen-activated protein kinase (MAPK) cascade for cell survival, dephosphorylation of TH phosphorylated at Ser31 (pSer31-TH) is very rare in living cells. Phosphorylation of TH at Ser8 has been shown in cultured rat pheochromocytoma PC12 cells and permeabilized bovine chromaffin cells after treatment with okadaic acid [57,66]. In contrast, no significant phenomena have been reported in cultured dopaminergic neurons and in vivo. These data suggest that TH regulation by Ser8 phosphorylation is not critical in the central nervous system.

## 4. Linkage of Tyrosine Hydroxylase Phosphorylation to Dopaminergic Pathology

As mentioned above, nigrostriatal TH protein is lost in PD and DRD. Ichinose et al. previously showed that Parkinsonian brains had very low levels of TH mRNAs in the substantia nigra compared with control brains, but no significant differences were found between schizophrenic and normal brains [68]. In addition, DRD patients have severely reduced (<3%) TH protein levels in the putamen [5,6]. These results suggest that TH protein levels in the nigrostriatal dopaminergic neurons are markedly decreased in both PD and DRD, but not in schizophrenia. Furthermore, Mogi et al. found that a decrease in total TH protein level in the striatum was greater than that in the total enzyme activity, as assessed by enzyme immunoassay [69]. This result suggests that upregulation of TH phosphorylation, which compensates decreased dopamine level, is linked to the reduction of nigrostriatal TH protein in PD. Intriguingly, we previously found that proteasomal inhibition leads to accumulation of pSer40-TH, which is ubiquitin-immunopositive, in nerve growth factor (NGF)-differentiated PC12D cells [70]. Moreover, Lewy bodies and Lewy neurites are pSer40-TH-immunopositive in PD [71]. TH protein, particularly phosphorylated TH, apparently forms intracellular aggregates easily [70,72]. In contrast, the dopamine- or biopterin-deficient state, which corresponds to PD or DRD, respectively, facilitates TH phosphorylation and leads to reduction of the total TH level in cultured cells [73,74]. The reduction of TH immunoreactivity can be observed in the midbrain and striatum of 6-pyruvoyl-tetrahydrobiopterin synthase-null and sepiapterin reductase-null mice, which are mouse models of tetrahydrobiopterin biosynthesis dysfunction [75,76]. Importantly, there is a difference in pathological features between PD and DRD, namely the presence or absence of abnormal protein accumulation. Here, a question arises. By which mechanism is nigral TH protein depleted, and does TH protein accumulate to form inclusions? Before we discuss the possible mechanism underlying the decrease in TH protein, let us take a brief look at protein degradation pathways.

## 5. Protein Degradation Pathways: Lysosome and Proteasome

**Figure 1:**
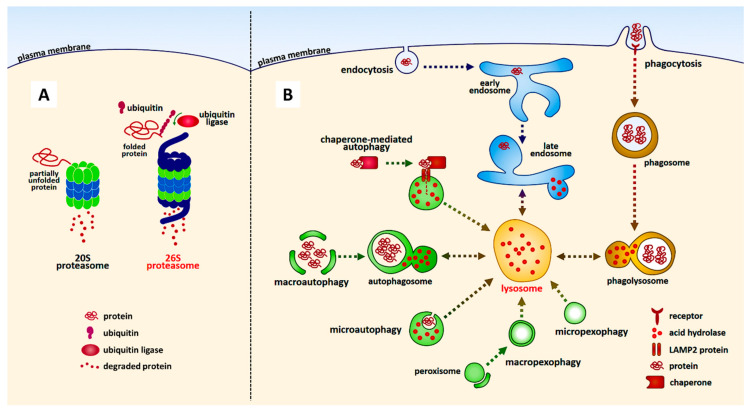
The representative protein degradation pathways in eukaryotic cells. Proteasome-dependent (**A**) and lysosome-dependent (**B**) pathways are shown. Note that not all the degradation pathways are illustrated in the figure.

The autophagy-lysosome and ubiquitin-proteasome pathways are the two main routes of protein and organelle clearance in eukaryotic cells [77] (Figure 1). Autophagy is a phenomenon in which cytoplasmic components are transported to lysosomes and degrade substrates, such as protein complexes and organelles, using lysosomal enzymes. There are various types of autophagy, namely selective and nonselective autophagy. The bulk degradation of cytoplasmic proteins or organelles is largely mediated by nonselective macroautophagy; a process generally referred to as autophagy. Selective macroautophagy employs the same core machinery used for nonselective macroautophagy. A small number of additional cargo-ligand-receptor-proteins serve to make the process selective [78,79,80,81,82,83]. Another well-known selective autophagy is chaperone-mediated. In chaperone-mediated autophagy, substrate proteins are selectively recognized by a cytosolic chaperone, the heat shock cognate protein of 70 kDa (hsc70) [84]. The interaction between the chaperone and the substrate in the cytosol targets the complex to the lysosomal membrane, where it binds to the lysosome-associated membrane protein type 2A (LAMP-2A), which acts as a receptor for this pathway [84,85]. In contrast, chaperone-unmediated autophagy is thought to function in the degradation of mitochondria.

Proteasomes are multiprotein complexes that predominantly degrade nuclear and cytosolic proteins. Most proteins are targeted for proteasomal degradation after being covalently modified with ubiquitin, which is conjugated through its carboxy terminus [86,87,88,89,90]. This reaction is called ubiquitination. Ubiquitin-protein conjugates are subsequently recognized and degraded by 26S proteasomes, which are multisubunit proteases found in the cytosol, perinuclear regions, and nucleus of eukaryotic cells [91]. The degradation products of 26S proteasomal catalysis are short peptide fragments and amino acids that can be recycled to produce new proteins. Simultaneously, polyubiquitin chains are released from targeted proteins and then disassembled by ubiquitin carboxy-terminal hydrolases to produce monomeric ubiquitin molecules that re-enter the ubiquitin-proteasome system, from which point they can contribute to the clearance of other abnormal proteins [92,93]. Failure of the ubiquitin-proteasome system is implicated in the pathogenesis of both sporadic and familial PD [22,23,24,94,95,96].

## 6. Ubiquitination and Proteasomal Degradation of Phosphorylated Tyrosine Hydroxylase

**Figure 2:**
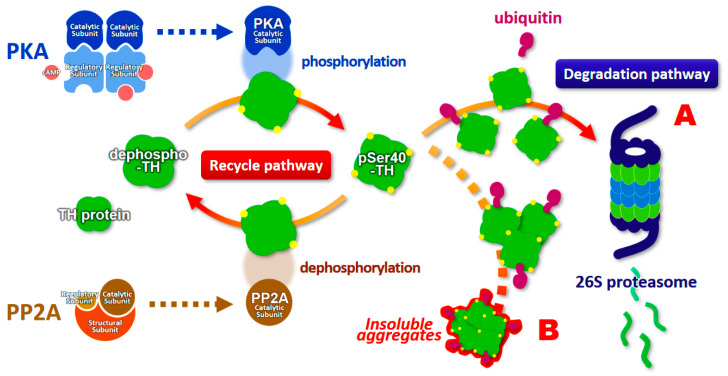
Schematic model of the proteasomal degradation of phosphorylated tyrosine hydroxylase (pSer40-TH). The degradation pathway is indicated in A, and the accumulation pathway to form insoluble aggregates is shown in B. pSer40-TH, tyrosine hydroxylase phosphorylated at serine 40 residue; cAMP, cyclic adenosine monophosphate; PKA, cAMP-dependent protein kinase; PP2A, protein phosphatase 2a.

Here, we introduce an evidence of the ubiquitination of phosphorylated TH and its proteasomal degradation by the ubiquitin-proteasome system, and discuss its possible physiological significance in PD and DRD (Table 1). First, Lazar et al. revealed that activated TH purified from bovine striatum showed decreased half-life at 50 °C [97]. They suggested that phosphorylation of TH could greatly increase the degradation rate of the enzyme in vivo. Several years later, Døskeland and Flatmark reported that human recombinant TH protein is ubiquitinated and degraded in the reticulocyte lysate system [98]. Subsequently, Urano et al. reported that recombinant human TH protein forms insoluble aggregates in the presence of tetrahydrobiopterin in vitro [99]. Recombinant TH is free from dopamine and presumably similar to phosphorylated TH [99]. We further revealed that 26S proteasomal inhibition leads to accumulation of TH phosphorylated at Ser40 (pSer40-TH), which are ubiquitin-positive, as well as formation of its insoluble inclusions in NGF-differentiated PC12D cells [70]. These observations support the novel pathway of proteasomal degradation of TH protein. The phenomenon of intracellular pSer40-TH insolubility unveiled the characteristics of pSer40-TH that it easily forms aggregates in living cells (Figure 2). Insight into the reduction of proteasomal activity in PD [94,95,96] further supports the evidence of the accumulation of pSer40-TH to form inclusion bodies in PD patients [71]. A publication by Nakashima et al. also showed the proteasomal degradation of the TH protein and evidence that phosphorylation of the N-terminal TH domain causes proteasomal degradation [100,101]. Carbajosa et al. also reported that short-term inhibition of proteasome increases the accumulation of ubiquitinated TH protein in PC12 cells and brainstem neurons [102], indicating that TH, especially phosphorylated TH, is ubiquitinated, resulting in its degradation by the ubiquitin-proteasome system.

**Figure 3:**
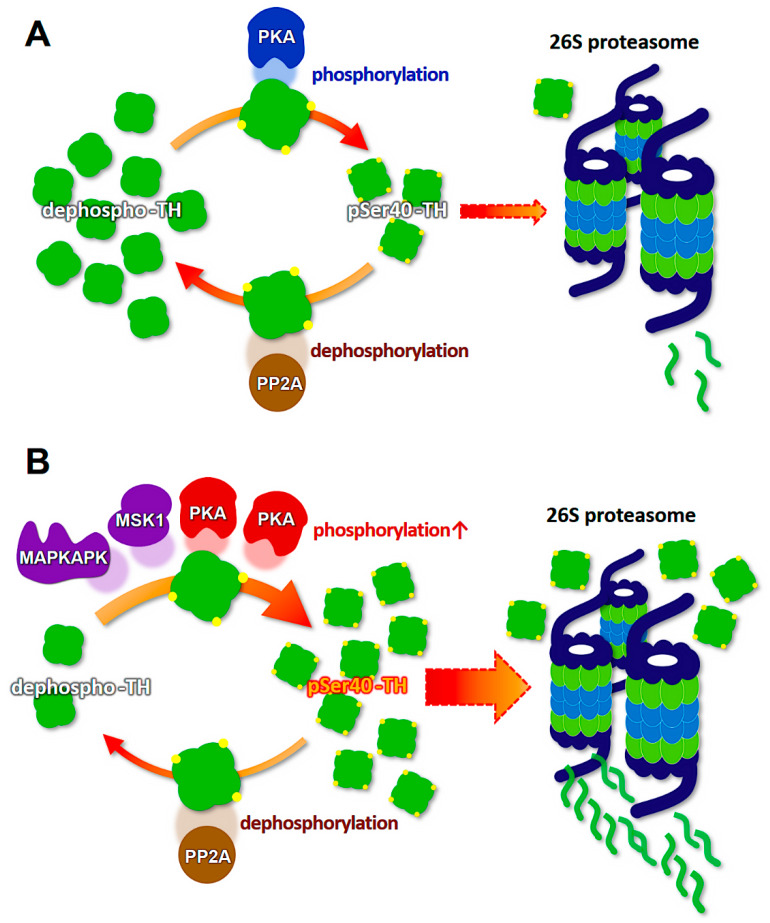
Schematic model of the molecular mechanism of the loss of tyrosine hydroxylase (TH) protein in the dopaminergic neurons. (**A**) A balanced state between the recycling TH protein and degrading TH protein. (**B**) Dopamine/biopterin deficient state activates PKA (red), and α-Synuclein aggregation presumably activates MAPKAPK and MSK1 (purple). Both activations accelerate TH phosphorylation (pSer40-TH), which is accompanied by proteasomal degradation. PKA, cAMP-dependent protein kinase; PP2A, protein phosphatase 2a. MAPKAPK, mitogen-activated protein kinase activated protein kinase; MSK1, mitogen- and stress-activated kinase 1.

What effect does the reduction of dopamine and biopterin levels have on the proteasomal degradation of phosphorylated TH in PD and DRD? Interestingly, dopamine and biopterin deficiencies lead to reduced total TH protein, which is caused by the degradation of pSer40-TH [74]. This pSer40-TH degradation was sensitive to MG-132, a 26S proteasome inhibitor [74], indicating a ubiquitin-proteasome system-mediated degradation. Salvatore et al. further revealed that knockout of dopamine transporter decreased dopamine content in the terminals of dopaminergic neurons, and this phenomenon was accompanied by the elevation of pSer40-TH and reduction of total TH protein [103]. Altogether, these data strongly suggest that phosphorylated TH protein is ubiquitinated to be degraded by the ubiquitin-proteasome system (Figure 2). Moreover, the lack of dopamine accelerates the proteasomal degradation of TH and its phosphorylation through PKA activation, resulting in the loss of TH protein and the negative spiral of TH depletion (Figure 3).

## 7. Modification of Tyrosine Hydroxylase Phosphorylation by α-Synuclein

α-Synuclein is a major component of Lewy bodies, and its deposition is a subset hallmark of neurodegenerative disorders, including PD, dementia with Lewy bodies (DLB), and multiple system atrophy, collectively referred to as synucleopathies. α-Synuclein was found in filamentous aggregates of Lewy bodies and Lewy neuritis [2,3], and the protein itself was first identified in 1993 as a nonamyloid β component of Alzheimer’s disease (AD) [104]. α-Synuclein isolated from DLB patients was phosphorylated [105]. α-Synuclein is degraded by proteasomes [106,107], and phosphorylated α-synuclein is ubiquitinated in α-synucleinopathy lesions [108], indicating that the ubiquitin-proteasome system degrades phosphorylated synuclein. Chaperone-mediated autophagy, which contributes to the degradation of intracellular proteins in lysosomes (Figure 1), also degrades α-synuclein [109,110,111].

**Figure 4:**
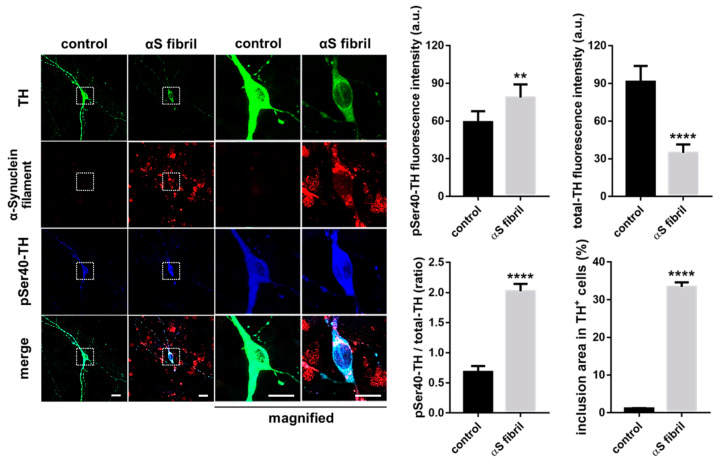
Exposure to α-Synuclein fibrils formed intracellular filamentous inclusions, which was accompanied by the acceleration of TH phosphorylation and the reduction of total TH protein in the cultured dopaminergic neurons in the presence of cycloheximide. Scale bars indicate 10 μm. The right columns show the quantified data (Student’s *t*-test, **** *p* < 0.0001, ** *p* < 0.01, *n* > 20). αS indicate α-Synuclein.

α-Synuclein itself seems to contribute to the maintenance of presynaptic function by participating in the assembly of the SNARE protein complex [112,113]. Furthermore, α-synuclein in the soluble form physically interacts with TH and maintains the level of phosphorylated TH in a PP2A-dependent manner [114,115,116], which suggests the possibility that α-synuclein monomer prevents excessive phosphorylation of TH by activating PP2A. Because the overexpression of wild-type or mutant human α-synuclein caused by the TH promoter did not result in the formation of pathological inclusions nor alter the behavior and sensitivity to 1-methyl-4-phenyl-1,2,3,6- tetrahydropyridine (MPTP) in C57BL/6 mice [117,118,119], factors other than α-synuclein itself may be associated with the neuronal degeneration of dopaminergic neurons. Thus, we hypothesize that not soluble α-synuclein monomers themselves, but oligomerized filaments and aggregates are associated with neurodegeneration. For instance, the failure of the ubiquitin-proteasome system in the substantia nigra in PD [94] presumably impairs the degradation of α-synuclein, which facilitates the formation of filamentous inclusions. Furthermore, dopamine-modified α-synuclein blocks chaperone-mediated autophagy [109,110], which induces abnormal intracellular accumulation α-synuclein in PD [120]. Plasma α-synuclein level in PD is higher than that in healthy controls [121], indicating possible reduction of protein degradation rate. Such aggregation of α-synuclein presumably potentiates TH phosphorylation and reduces total TH protein [116,122,123]. Indeed, we revealed that the formation of intracellular aggregations of filamentous α-synuclein led to a decrease in the total TH protein levels with increased pSer40-TH in cultured dopaminergic neurons (Figure 4). α-Synuclein activates stress-related protein kinases to potentiate TH phosphorylation at serine 40, suggesting the possible mechanism of pSer40-TH elevation by α-synuclein aggregation [52,124,125]. The α-synuclein-induced abnormal upregulation of TH phosphorylation, combined with the reduction of gene transcription by aging and aging-related disorders [126,127,128,129], results in the acceleration of pSer40-TH degradation to reduce total TH protein (Figure 3).

## 8. Novel Therapeutic Targets for α-Synuclein Propagation

Previously, when the molecular mechanism of PD pathogenesis was not well understood, PD patients have been prescribed a dopamine precursor, L-3,4-dihydroxyphenylalanine (L-DOPA) [130]. Oral administration of L-DOPA led to partial improvement of PD symptoms; however, L-DOPA exerts side effects, such as nausea and vomiting, which had been able to be attenuated by slowing the increases in the daily dose [130,131]. Second, after prolonged treatment with L-DOPA, as many as 72% of Parkinsonism patients will suffer from movement disorders. These disorders consist of uncontrollable facial movements, namely grimacing, tongue protrusion, and chewing motions [131,132]. A third side effect is a loss of blood pressure upon standing; approximately 33% of patients have shown this effect [131,132]. This problem tends to disappear in patients receiving the drug for a sufficiently long period. Although L-DOPA has such uncomfortable side effects [133], it is still useful for treating PD and DRD [134] and often used in combination with carbidopa, which inhibits peripheral metabolism of L-DOPA. Therefore, L-DOPA is expected to be used in combination with novel therapeutic agents.

Recently, the propagation of α-synuclein is focused on PD pathogenesis [135,136]. Accumulation of propagated α-synuclein results in synucleinopathies, including PD, DLB, and multiple system atrophy [137]. As introduced in Section 7, the aggregation of propagated α-synuclein alters TH phosphorylation, which is accompanied by the proteasomal degradation of pSer40-TH to decrease total TH protein (Figure 4). Furthermore, α-synuclein contributes to the fibrilization of amyloid-β and tau [138], which are two critical proteins in AD, suggesting a central role of α-synuclein toxicity in neurodegeneration. Thus, α-synuclein uptake into living neuronal cells is critical for the pathogenesis of synucleinopathies. Then, how can we prevent α-synuclein propagation and its uptake into dopaminergic neurons?

Various molecular mechanisms are expected to be involved in α-synuclein uptake; for example, mechanisms related to the α3-subunit of Na^+^/K^+^-ATPase [139], neurexin [140,141], flotillin [142], and particular endocytic pathways [143]. Very recently, we showed that fatty acid-binding protein 3 (FABP3) is critical for α-synuclein uptake into dopaminergic neurons [144] and enhancement of α-synuclein spreading [145]. FABP3 is also essential in 1-methyl-4-phenylpyridinium (MPP^+^)-induced morphological abnormality, mitochondrial dysfunction and neurotoxicity [144]. The injury induced by MPTP or its metabolite, MPP^+^-, to dopaminergic neurons of the nigrostriatal pathways of nonhuman primates has been an important model for parkinsonism as well as dystonia [146,147,148,149]. These data suggest that FABP3 is a potential therapeutic target in synucleinopathies that can act by preventing α-synuclein uptake into dopaminergic neurons. Intriguingly, FABP3 ligand, which we have recently synthesized, inhibits α-synuclein oligomerization in PD mouse models [150,151]. These data suggest that FABP3-targeting ligands are potential therapeutic candidates for synucleinopathies.

Intriguingly, serum FABP3 level is increased in PD [152]. Although cerebrospinal fluid (CSF) is the nearest body fluid to the cerebral parenchyma as a biomarker of the central nervous system, the method of obtaining CSF is invasive and painful. Serum or plasma derived from blood is an ideal body fluid that can be used for screening of biomarker levels, as it is easily obtainable, and its collection process causes minimal discomfort. Previously, plasma levels of phosphorylated tau [153], amyloid-β (1-40/1-42) [154,155,156,157,158,159], and α-synuclein [121] have been studied for their potential to predict or diagnose AD and PD. The average value of each biomarker changes significantly; however, it is not sufficient to accurately predict specific disorders because some patients with AD or PD show lower plasma amyloid-β and α-synuclein levels than those of healthy controls. Therefore, novel diagnostic tools will be required. When we can predict PD at the very early stage and prevent the interaction of α-synuclein and FABP3 before the onset of PD, accumulation of the protein and its-induced neurotoxicity will be abolished. We will further study the pharmacologic action and molecular mechanism of FABP3-targeted compounds to prevent dopaminergic neurons from α-synuclein propagation and to promote neuronal survival [50,160,161,162,163,164], and we will develop a diagnostic method for predicting PD at the very early stages.

## 9. Conclusions

**Figure 5:**
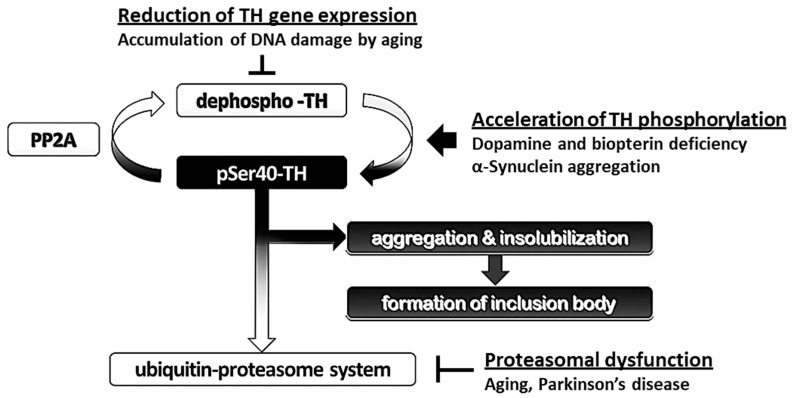
Schematic model for the pathways of degradation and accumulation of tyrosine hydroxylase (TH) protein. The ubiquitin-proteasome system degrades phosphorylated TH at serine 40 (pSer40-TH). Otherwise, pSer40-TH is accumulated in the proteasomal deficient state and forms insoluble aggregates.

It is unclear why dopaminergic neurons preferentially degenerate in PD and DRD. Many factors may contribute to this, including mitochondrial dysfunction [165,166], oxidative stress, decreased glutathione content [167], increased iron levels [168], and production of oxygen radicals through the combination of dopamine and tetrahydrobiopterin [169,170]. Here, we present clues to understanding this selective degeneration of dopamine-containing neurons, which are sensitive to dopamine/biopterin deficiency and α-synuclein invasion. The consequent elevation of TH phosphorylation is followed by the degradation of pSer40-TH by the ubiquitin-proteasome system. Interestingly, proteasomal inhibition results in TH aggregation, whereas choline acetyltransferase does not show such aggregations [70]. Owing to such characteristics of TH protein to aggregate and be degraded easily, especially pSer40-TH, handling phosphorylated TH is somehow tricky for dopaminergic neurons. The formation of insoluble inclusions of pSer40-TH further reduces cytoplasmic operable TH. We suggest that the negative spiral mechanism of TH phosphorylation-induced degradation is involved in the loss of nigrostriatal TH protein in PD and DRD (Figure 5).

In the present article, we have reviewed the molecular mechanism of the loss of mesencephalic TH protein in PD and DRD. We conclude that the ubiquitin-proteasome system participates in the degradation of phosphorylated TH. The mechanism of ubiquitin-proteasome-linked dopaminergic pathogenesis might help explain the dopaminergic neuron-selective loss of TH protein in PD and DRD. These insights may lead to more focused efforts to develop therapeutics and strategies to prevent the onset of neurodegeneration in PD and DRD.

## Figures and Tables

**Table 1:** Advances of the study for the ubiquitination and proteasomal degradation of phosphorylated tyrosine hydroxylase protein (original articles).

Evidence	Year	Reference
Activated tyrosine hydroxylase purified from bovine striatum decreases its thermal stability	1981	[97]
Human recombinant TH protein is ubiquitinated and degraded in the reticulocyte lysate system	2002	[98]
Recombinant human TH forms insoluble aggregates in the presence of tetrahydrobiopterin	2006	[99]
Proteasomal inhibition accumulates ubiquitinated TH protein phosphorylated at ^40^Ser to form insoluble aggregates in NGF-differentiated PC12D cells	2009	[70]
Phosphorylation of the N-terminal domain of tyrosine hydroxylase triggers proteasomal digestion	2011	[100]
Short-term inhibition of proteasome increases the accumulation of ubiquitinated TH protein in PC12 cell and brainstem neurons	2015	[102]
Dopamine or biopterin deficiency potentiates phosphorylation at ^40^Ser and ubiquitination of TH protein to be degraded by the ubiquitin proteasome system	2015	[74]
Inhibition of USP14 to activate proteasome decreases TH protein phosphorylated at ^19^Ser	2016	[101]
Dopamine transporter-deficiency increases TH phosphorylation and decreases TH protein in striatum and nucleus accumbens	2016	[103]

TH, tyrosine hydroxylase; NGF, nerve growth factor; USP14, Ubiquitin-specific protease 14.

## Abbreviations

AD	Alzheimer’s disease
AP-1	Activator protein 1
CaMKII	Calcium/calmodulin-dependent protein kinase II
cAMP	Cyclic adenosine monophosphate
CMA	Chaperone-mediated autophagy
CSF	Cerebrospinal fluid
DLB	Dementia with Lewy bodies
DRD	Dopa-responsive dystonia (Segawa disease)
ERK	Extracellular signal-regulated kinase
FABP	Fatty acid-binding protein
GTP	guanosine triphosphate
GCH1	GTP cyclohydrolase 1
Hsc70	Heat shock cognate protein of 70 kDa
L-DOPA	L-3,4-dihydroxyphenylalanine
LAMP	Lysosome-associated membrane protein
LRRK2	Leucine-rich repeat kinase 2
MAPK	mitogen-activated protein kinase
MAPKAPK	Mitogen-activated protein kinase activated protein kinase
MPP+	1-methyl-4-phenylpyridinium
MPTP	1-methyl-4-phenyl-1,2,3,6-tetrahydropyridine
MSK1	Mitogen- and stress-activated kinase 1
NGF	Nerve growth factor
Nurr1	Nuclear receptor related-1
PD	Parkinson’s disease
PP2A	Protein phosphatase 2a
pSer40-TH	Tyrosine hydroxylase phosphorylated at Ser40
SNpc	Substantia nigra pars compacta
SRF	Serum-responsive factor
TH	Tyrosine hydroxylase
VTA	Ventral tegmental area
